# Ameliorative properties of aqueous extract of *Ficus thonningii* on erythrocyte osmotic fragility induced by acetaminophen in *Rattus norvegicus*

**Published:** 2013

**Authors:** Victor Masekaven Ahur, Yahaya Adenkola Adenkola, Saganuwan Alhaji Saganuwan, Job Terungwa Ikye-Tor

**Affiliations:** 1 *Department of Veterinary Physiology, Pharmacology and Biochemistry, College of Veterinary Medicine, University of Agriculture, Makurdi, Nigeria;*; 2 * Division of Livestock Services, Benue State Ministry of Agriculture and Natural Resources, Makurdi, Nigeria.*

**Keywords:** Acetaminophen, Erythrocytes, *Ficus thonningii*, Osmotic fragility

## Abstract

*In vitro* antioxidant and erythrocyte protecting activities by aqueous extract of *Ficus thonningii *leaves on blood cells were studied in acetaminophen treated rats. The extract was safe at limit dose of 5000 mg kg^-1^ body weight. The extract demonstrated dose dependent antihemolytic effect at dose levels between 50 and 200 mg kg^-1^ body weight. The lowest antihemolytic effect was observed at dose level of 200 mg kg^-1^ body given the lowest percentage hemolysis of 10.53 ± 1.76%, whereas the highest percentage hemolysis at dose level of 50 mg kg^-1^ was 29.02 ± 7.45%. Hematology revealed erythrocytosis at dose levels of 100 and 200 mg kg^-1^ body weight. Hyperglobinemia and lymphocytopenia were observed at dose levels of 100 mg kg^-1^ and 200 mg kg^-1^, respectively. The extract effectively showed scavenging activity on a stable oxidative radical diphenylpicrylhydrazyl (DPPH) and a significant ferric reducing antioxidant power (FRAP) activity. The plausible erythrocyte membrane protective effect may be due to its free radical scavenging activity and hence the extract can be used to improve hematological parameters and ameliorate oxidative stress.

## Introduction

*Ficus thonningii* (Blume) belonging to the family Moraceae commonly known as wild fig is a large economic tree indigenous to savannah part of Nigeria and the leaves have been of interest to researchers because of its increased use in folkloric medicine.^1^^,^^2^ It is widely used for treatment of sore throat, colds, diarrhea, jaundice, malaria fever, dysgalactia,^2^ gonorrhea and respiratory infections.^3^ It has been observed to possess analgesic and anti-inflammatory properties,^4^^,^^5^ antimicrobial activity^4^^,^^6^ and reduced intestinal motility.^7^ *Ficus thonningii* leaves constitute many primary and secondary metabolites such as anthraquinone, glycosides, steroids, unsaturated steroids, aglycones, tannins, saponins, flavonioids, triterpenes and alkaloids which play an important and varied pharmacological actions that contribute to the therapeutic claims of the plant.^6^ It is used by traditional practitioners of some communities in Nigeria for treating malaria fever, liver disorders, jaundice and other related diseases.^8^ Our previous investigations revealed that ethyl acetate fraction of *F. thonningii* has antihemolytic and hematinic properties by probably stabilizing the membrane of erythrocytes in rats.^8^ The growing interest in herbal medicine demands information on various plant preparations used in the treatment of diseases.^9^ Therefore, scientific evaluation of these medicinal plants is important to the discovery of novel drugs. 

The present study investigated the effects of aqueous fraction of *F. thonningii* (AQFT) leaves on erythrocytes membrane integrity and hematological parameters of acetaminophen induced oxidative stress in rats. 

## Materials and Methods

**Collection and identification of plant material.** Fresh leaves of *F. thonningii* were collected from Kyado village, Mbazum ward of Ukum Local Government Area of Benue State, Nigeria in May, 2009 and identified by a plant taxonomist of Bioresources Development and Conservation Programme, Nsukka, Nigeria. 

**Extraction and fractionation of** ***F. thonningii*** **leaves. **The leaves were air dried at room temperature to a constant weight and pulverized using a laboratory miller. The powder was placed in an air-tight container and stored at room temperature until required for use. About 346.20 g of the pulverized leaves were defatted with absolute n-Hexane and then extracted with 70 % methanol using Soxhlet apparatus. Removal of solvents *in vacuo* at 40 ˚C, respectively yielded 13.98 g and 17.81 g of n-Hexane and methanol extracts of *F. thonningii*. The methanol extract was subjected to partitioning with ethyl acetate and distilled water to yield 3.87 g (1.10%) of ethyl acetate fraction (EAFT) and 13.79 g (4.0%) of AQFT. The fractions were stored at 4 ˚C until required for use. The aqueous fraction was used for the study.

**Experimental animals.** All the animals used for this study were obtained from a stock bred at Olusegun Obansanjo College of Health Sciences, Benue State University, Makurdi, Nigeria. The animals were kept in cages and housed at Department of Veterinary Physiology, Pharmacology and Biochemistry, College of Veterinary Medicine, University of Agriculture, Makurdi, for a 2-weeks period for acclimatization and during this period the animals were accustomed to routine handling. The rats were fed standard commercial rat pellets (UAC Grand Cereals Ltd., Jos, Nigeria) and water was provided *ad libitum*. The animals were used in accordance with the guidelines and recommendations of the ethic committee on the use of animals for research of the University of Agriculture, Makurdi with permit number P/No. 2009006. 

**Median lethal dose estimation. **Median lethal dose (LD_50_) was estimated using revised up-and-down procedure (UDP) as described by US EPA^10^ and Gosh^11^ using 5000 mg kg^-1^body weight test limit dose (volumes more than 2.0 mL were divided into two parts and administered with in a time frame of 30 to 45 min). Then, ^1^/_100_^th^, ^1^/_50_^th^ and ^1^/_25_^th^ of the limit dose (50 mg kg^-1^, 100 mg kg^-1 ^and 200 mg kg^-1^, PO), respectively, were adopted for erythrocyte osmotic fragility test and hematological parameters determination.

**Experimental design. **A total number of 30 animals of either sex weighing 125.57 ± 2.78 g were divided into five groups of six animals each. Groups 1 and 2 which served as normal and negative controls were administered 5 mg kg^-1 ^body weight of distilled water. Groups 3, 4 and 5 were treated orally with AQFT at dose levels of 50, 100 and 200 mg kg^-1 ^body weight, respectively. All the animals were treated every 12 hr with the extract for 5 consecutive days. An hour after the last treatment on the 5^th^ day, all groups except group 1, were administered a single 2000 mg kg^-1^ body weight acetaminophen (May and Baker PLC, Lagos, Nigeria), orally. After 18 hr of acetaminophen treatment, 2.0 mL of blood sample was collected from each rat through the retro bulbar plexus using microcapillary tubes puncture and placed into ethylene diamine tetra-acetic acid (EDTA; Chengdu Rich Science Industry Co., Sichuan, China) bottles for erythrocyte osmotic fragility test.^12^ Hematological parameters, total red blood cell count, total white blood cell count, packed cell volume (PCV) and hemoglobin con-centration, were determined using the method described by Schalm *et al*.^13^ The mean corpuscular volume (MCV), mean corpuscular hemoglobin (MCH) and mean corpuscular hemoglobin concentration (MCHC) values were calculated from the values of hemoglobin concentration, packed cell volume and total erythrocyte count.^13^ Differential white blood cells were determined using the method of Cole.^14^

***In vitro*** **antioxidant activity of AQFT using DPPH (1, 1-diphenyl-2-picrihyrazyl). **The ability of the AQFT to scavenge DPPH radical was determined according to the method described by Mensor *et al*.^15^ Each 2.0 mL of the extract containing 10, 50, 100, 200 and 400 μg mL^-1 ^was mixed with 1.0 mL of 0.5 mM DPPH (in methanol) and allowed to react in the dark at room temperature for 30 min. The absorbance of the resulting mixture was measured at 517 nm. The experiments were done in triplicate. The percentage antioxidant activity (AA %) was calculated as follows; 

$\mathrm{Antioxidant activity}\left(\%\right)=100\times \frac{\left[({\mathrm{Abs}}_{\left(\mathrm{sample}\right)}-{\mathrm{Abs}}_{\left(\mathrm{blank}\right)})\times 100\right]}{{\mathrm{Abs}}_{\left(\mathrm{control}\right)}}$

Methanol (1.0 mL) plus 2.0 mL of the extract was used as blank while 1.0 mL of 0.5 mM DPPH plus 2.0 mL methanol was used as negative control. The DPPH free radical scavenging activity of ascorbic acid (Sigma-Aldrich, St. Louis, USA) was used as reference standard.

**Ferric reducing antioxidant power assay of AQFT. **The total antioxidant potential of the extract was determined using FRAP assay of Benzie and Strain.^16^ The FRAP reagent was prepared by mixing acetate buffer (Alpha Chemika, Mumbai, India) of pH 3.6, solution “X” and 20 mM FeCl_3_ solution (Sigma- Aldrich, St. Louis, USA) in proportions 10:1:1 (v/v/v), respectively. Solution “X” was prepared by mixing 10 mM 2, 4, 6-tri(2-pyridyl)-s-triazine (TPTZ, Sigma- Aldrich, St. Louis, USA) and 40 mM HCl (Bredox BV, Rotterdam, Netherlands). Fresh FRAP reagent used for the analysis was warmed to 37 ˚C prior to use. Then 10-400 µM (100 µL) of AQFT was added to 3 mL (3000 µL) of the working FRAP reagent. The absorbance (593 nm) was measured at immediately after vortexing. Thereafter, samples were incubated at 37 ˚C in a water bath and absorbance measured again after 4 min. Ascorbic acid (100-1000 µM) was assayed and the FRAP of AQFT (sample) was calculated as follows;

$\mathrm{FRAP}=\frac{{∆\mathrm{Abs}}_{\left(\mathrm{sample}\right)}\mathrm{from 0 to 4}\min\times \mathrm{FRAP value of standard}}{{∆\mathrm{Abs}}_{\left(\mathrm{standard}\right)}\mathrm{from 0 to 4 min}}$

**Statistical analysis. **IBM SPSS software (IBM, Armonk, USA) was used for the analysis. All data were expressed as mean ± SEM and analyzed using one way analysis of variance (ANOVA) at 5% level of significance. Least significant difference (LSD) was used to detect differences among the treatment groups.^17^^,^^18^

## Results

Aqueous fraction of *F. thonningii* leaf extract did not produce any sign of acute toxicity at limit dose of 5000 mg kg^-1 ^body weight for a period of 14 days. Hence, the extracts can either be classified as category 4 or category 5 medicinal plant product, respectively.^19^^,^^20^

**Fig. 1:**
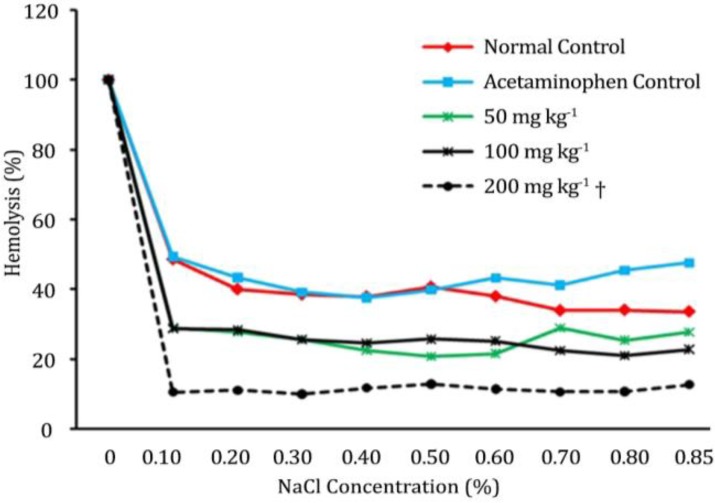
Effect of AQFT on erythrocyte osmotic fragility in acetaminophen-treated rats; (^†^ values are significantly different compared to normal and acetaminophen control groups,* p* < 0.05).

Effect of AQFT on erythrocyte osmotic fragility in acetaminophen-treated rats. The maximum hemolysis was observed in the normal control (group 1) at 0.1% sodium chloride concentration having a value of 48.54 ± 7.30% which decreased as the concentration of NaCl increased; given a value of 35.58 ± 8.62% at 0.85% of NaCl concentration. However, at 0.85% concentration the group 5 rats treated with 200 mg kg^-1 ^of AQFT had significantly (*p* < 0.05) lowered erythrocyte fragility given the lowest percentage hemolysis of 10.53 ± 1.76%, while the highest percentage hemolysis was obtained at the same concentration in the group treated with 50 mg kg^-1 ^of AQFT given a value of 29.02 ± 7.45% (Fig. 1). Generally, the percentage hemolysis of the control groups (normal and acetaminophen treated groups) were significantly (*p* < 0.05) higher as compared to the percentage hemolysis observed in the groups treated with graded doses (50, 100 and 200 mg kg^-1^, PO) of AQFT (Fig. 1). 

**Effect of AQFT on hematological parameters of acetaminophen-treated rats. **The recorded values of hemoglobin and total leucocyte count were significantly (*p* < 0.05) lowered in the groups treated with acetaminophen alone (Group 2) given values of 12.23 ± 0.19% and 7.12 ± 0.53% × 10^9^ per liter, respectively. A significant (*p* < 0.05) increase in the values of total erythrocyte count were observed in the groups of rats treated with 100 and 200 mg kg^-1 ^of AQFT with resultant values of 7.20 ± 0.15 × 10^12^ per liter and 7.16 ± 0.23 × 10^12^ per liter, respectively. The highest values of 64.33 ± 4.39% and 63.50 ± 2.23% recorded in the groups treated with 100 mg kg^-1^ (Group 4) and 200 mg kg^-1^ (Group 5), respectively did not differ significantly (*p* > 0.05) in comparison with the control values (Table 1).

**Fig. 2:**
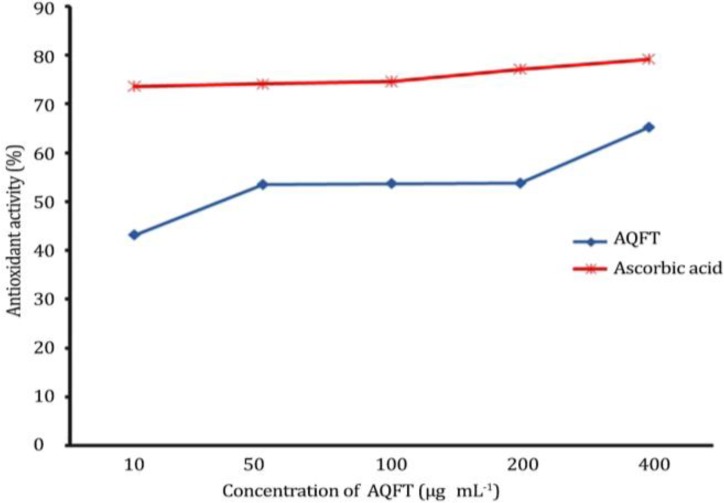
Antioxidant activity of AQFT as compared with ascorbic acid using DPPH method.

***In vitro*** **radical scavenging activity of AQFT using DPPH. **The *in vitro* antioxidant radical scavenging activity of AQFT using DPPH yielded results comparable with that of ascorbic acid. The AQFT and ascorbic acid scavenged DPPH in a dose dependent manner. The higher the concentration of the extract, the better the percentage of its antioxidant activity. At a maximum concentration of 400 µm mL^-1^, the crude AQFT has a percentage antioxidant activity of 65.24 ± 1.36 comparable to 79.21 ± 1.60 of ascorbic acid, the reference compound (Fig. 2).

**Table 1:** Effects of AQFT leaves on hematological parameters of acetaminophen-treated rats. Data are presented as mean ± SEM, (n = 6).

				**Dose of AQFT + Acetaminophen **		
**Index**		**Control**	**Acetaminophen**	**50 mg kg** ^-1^	**100 mg kg** ^-1^	**200 mg kg** ^-1^
**Hemoglobin (g dL** ^-1^ **)**		12.52 ± 0.29	12.23 ± 0.19^a^	12.65 ± 0.33	13.23 ± 0.38^b^	12.65 ± 0.43
**Packed cell volume (%)**		42.22 ± 1.00	41.35 ± 0.86	41.32 ± 1.27	44.15 ± 1.35	42.05 ± 1.42
**Mean corpuscular volume (fL)**		63.58 ± 1.23	62.73 ± 0.86	59.70 ± 0.47	61.25 ± 0.91	58.75 ± 0.95
**Mean corpuscular hemoglobin (pg)**		18.85 ± 0.25	18.57 ± 0.24	18.30 ± 0.17	18.38 ± 0.24	17.65 ± 0.18
**Mean corpuscular hemoglobin concentration (g dL** ^-1^ **)**		29.68 ± 0.38	29.60 ± 0.24	30.65 ± 0.32	29.97 ± 0.11	30.08 ± 0.32
**Red blood cells (×10** ^6^ ** μL** ^-1^ **)**		6.65 ± 0.18^a^	6.60 ± 0.14^a^	6.92 ± 0.22	7.20 ± 0.15^b^	7.16 ± 0.23^b^
**White blood cells (×10** ^3^ ** μL** ^-1^ **)**		11.53 ± 1.20	7.12 ± 0.53^a^	8.08 ± 1.08	11.53 ± 0.35^b^	10.10 ± 1.13^b^
**Neutrophils (%)**		30.17 ± 3.17	35.33 ± 5.02	35.67 ± 4.53	29.83 ± 3.81	35.33 ± 3.67
**Lymphocytes (%)**		62.50 ± 3.84	59.67 ± 4.29	60.50 ± 4.88	64.33 ± 4.39	63.50 ± 2.32

a,b Values with different alphabet superscripts on the same row are significantly different at *p *< 0.05.

**Fig. 3:**
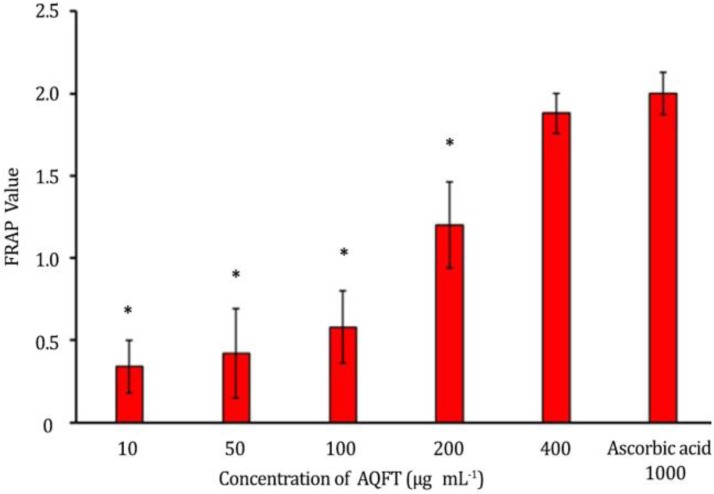
Ferric reducing antioxidant power of AQFT compared with ascorbic acid; (* values are significantly different compared to the reference drug ascorbic acid,* p* < 0.05).

**Ferric reducing antioxidant power assay of AQFT.** Figure 3 shows the antioxidant capacity of AQFT using FRAP. The antioxidant capacity of AQFT increased in concentration dependent fashion from 0.34 ± 0.16 µM at 10 μg mL^-1 ^to 1.88 ± 0.12 µM at 400 μg mL^-1^, respectively. The FRAP value of ascorbic acid at concentration of 100 to 1000 µM is 2.0.^16^ However, at 400 μg mL^-1^, FRAP of AQFT (1.88 ± 0.12 µM) was almost similar to that of ascorbic acid (reference standard).^16^

## Discussion

The result of the present investigation showed that AQFT is safe since there was no sign of acute toxicity observed on the animals administered oral test limit dose of 5000 mg kg^-1 ^of the extract. Our finding is in agreement with the earlier report of Ahur *et al. *who reported that ethyl acetate fraction of the methanolic extract of *F. thonningii* is safe.^8^ The efficacy of any membrane stabilizing agent is dependent on its ability to either reduce the harmful effects or restore the normal physiology of the cell membrane that has been disrupted by a toxicant.^21^ Pre-treatment with oral dose of AQFT at 50, 100 and 200 mg kg^-1 ^dose dependently reduced the percentage hemolysis of the erythrocytes in hypotonic saline in comparison to the normal and negative controls. The extract at 200 mg kg^-1^ exhibited a better membrane protective ability with relatively lower fragiligram. Our findings agree with the report of Nigeria Natural Medicine Development Agency (NNMDA) and Orwa *et al.* indicating that *F. thonningii *is among a combination of plants used in the treatment of various diseases characterized with anemia, jaundice and fatigue.^2^^,^^3^ Our findings in this study are also in concordance with the report of Ahur *et al.* indicating that ethyl acetate extract of *F. thonningii* has a dose dependent antihemolytic activity in acetaminophen-induced erythrocyte membrane damage.^8^ Antioxidants have been used to protect the membrane integrity of erythrocytes membrane during oxidative stress,^22^^,^^23^ and herbal preparations possessing antioxidant property are also capable of stabilizing the red blood cell membrane.^21^^,^^24^ The erythrocyte membrane protective ability of this extract may be due to the presence of flavonoids,^6^ and flavonoids have been reported to have antioxidant property.^25^ Reportedly, flavonoids exert profound effects capable of stabilizing the erythrocyte membrane,^26^ and are also known to scavenge on free radicals.^27^ Substances like glutathione, tocopherol and proactive enzyme have already been identified as anti-oxidants capable of preventing oxidation of susceptible substrate.^28^^,^^29^ Acetaminophen is known to cause liver damage by formation of excess, highly reactive metabolites (N-acetyl-p-benzoquinoneimine)^30^^,^^31^ causing depletion of glutathione and invariably resulting to oxidative stress^32^^,^^33^ weakening of erythrocyte membrane and finally lysis of erythrocytes,^34^ hence, higher fragiligram as seen in control group in this study. Erythrocyte is susceptible to oxidative damage due to the high polyunsaturated fatty acid content of the membrane and the high cellular concentration of oxygen and hemoglobin, which are all powerful promoters of oxidative processes.^35^ The AQFT effectively scavenged DPPH, a stable free radical and it also exhibited significant FRAP activity similar to the reference drug ascorbic acid. Therefore, the possible mechanism of antihemolytic activity of AQFT may involve prevention of free radicals that would cause oxidative stress and cellular damage.^29^

The animals in group 4 and 5 showed significant improvement in total erythrocytes count compared to the normal and acetaminophen groups. This finding is corroborated by the earlier findings of Ahur *et al.*^8^ that ethyl acetate fraction of *F. thonningii* improves hematological parameters of albino rats.^8^ The extract may have erythropoietic ability which is at variance with the report of Coker *et al. *who indicated that *F. thonningii* lacks hemopoietic tendency but more study is still needed in this direction to elucidate this fact.^36^ The lymphocytosis observed in our study agrees with the report of Aniagu *et al. *who observed a significant increase in lymphocyte levels of rats administered extract of *F. thonningii*.^37^ Leukopenia seen in the acetaminophen group may be attributed to the oxidative stress caused by the insult (acetaminophen) on the rat hepatocytes.

Acetaminophen (N-acetyl-para-aminophen) is a well-known antipyretic/analgesic agent, although safe at therapeutic doses, overdose can produce severe hepatic, renal and hematological pathologies when high doses are given.^38^^,^^39^ Acetaminophen toxicity is through the formation of excess, highly reactive metabolites (N-acetyl-p-benzo-quinoneimine) invariably resulting to oxidative stress,^39^^,^^40^ hence, its deleterious effects on cell lipids, proteins, and plasma membrane damage.^32^^,^^33^ The mechanism of damage involves lipid peroxidation, membrane protein cross linking and fragmentation (lysis).^41^^,^^42^ Free radicals have been reported to cause red blood cell lysis^34^ due to lipid per-oxidation,^43^ increased membrane deformability^44^^,^^45^ and membrane protein cross linking and fragmentation.^46^

In conclusion, aqueous fraction of *F. thonningii* leaves is safe at 5000 mg kg^-1^. It has protective ability on integrity of erythrocyte membrane in addition to improving hematological parameters and also possesses free radical scavenging activity *in vitro.*
